# Molecular and clinical characterization of Galectin‐9 in glioma through 1,027 samples

**DOI:** 10.1002/jcp.29309

**Published:** 2019-10-14

**Authors:** Feng Yuan, Haolang Ming, Yingshuai Wang, Yihan Yang, Li Yi, Tao Li, Haiwen Ma, Luqing Tong, Liang Zhang, Peidong Liu, Jiabo Li, Yu Lin, Shengping Yu, Bingcheng Ren, Xuejun Yang

**Affiliations:** ^1^ Department of Neurosurgery Tianjin Medical University General Hospital Tianjin China; ^2^ Laboratory of Neuro‐Oncology Tianjin Neurological Institute Tianjin China; ^3^ Department of Internal Medicine III, University Hospital Munich Ludwig‐Maximilians‐University Munich Munich Germany

**Keywords:** checkpoint inhibitors, Galectin‐9, glioblastoma multiforme, immune response, tumor‐associated macrophages

## Abstract

In recent years, research on glioma immunotherapy have grown rapidly. However, the autoimmune‐like side effects that are caused by blocking immunological checkpoints hinder their clinical application in gliomas currently. Galectin‐9, a ligand for T‐cell immunoglobulin mucin 3, has shed a new light on the treatment of malignant glioma. However, the potential mechanism of Galectin‐9 is still under discussion. In this study, first, we methodically gathered 1,027 glioma patients with RNA‐seq and 986 patients with survival data to explore the role and mechanism of Galectin‐9 in gliomas. Second, we analyzed glioma samples from 50 patients in the Department of Neurosurgery, Tianjin Medical University General Hospital. Finally, we found that Galectin‐9 was strongly upregulated in glioblastoma multiforme compared with normal brain tissues and lower‐grade glioma. Patients with Galectin‐9 overexpression had a significantly shorter overall survival. Moreover, the tissue microarray data displayed that the expression of Galectin‐9 in the core of tumor is higher than that in the border and was correlated with the shorter survival in glioma patients. Galectin‐9 is more highly expressed in the mesenchymal subtype of glioblastoma multiforme than in the other subtypes. Simultaneously, Galectin‐9 was closely associated with the immune response and lymphocyte activation, especially T‐cell activation. To further determine the underlying role of Galectin‐9 in the immune response, we selected seven immune metagenes. Through cluster analysis and correlation analysis, we discovered that Galectin‐9 was highly correlated with immune checkpoint molecules and M2 tumor‐associated macrophages. In summary, Galectin‐9 serves as a potential therapeutic target to treat glioblastoma multiforme.

## Graphical abstract

In this study, we evaluated the expression status of Galecin‐9 and its associated biological processes and prognosis by analyzing RNA‐seq and clinical data from TCGA and CGGA databases, with the hope of obtaining a comprehensive understanding of Galectin‐9 and new findings in its use for the treatment of glioblastoma multiforme (GBM)

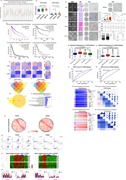

## 1 INTRODUCTION

Nearly 80% of gliomas are primary brain tumors, and glioblastoma multiforme (GBM) is the most invasive and incurable type. Genomic analysis has been used to divide GBM into four major molecular subtypes: neural, proneural, classical, and mesenchymal (Lapointe, Perry, & Butowski, 2018). These subtypes are closely related to the excessive activation of specific signaling pathways and prognosis of the patients. The current standard of treatment for newly diagnosed GBM patients is surgical resection, concurrent chemoradiotherapy, and adjuvant chemotherapy (Weller et al., 2014). Despite these treatments, GBM eventually relapses in almost all patients. So, there is still a long way to go in method upgradation in glioma treatment (Wen & Reardon, 2016).

Cancer immunotherapy refers to a treatment that increases tumor‐specific adaptive immunity other than directly targeting tumor cells (Khalil, Smith, Brentjens, & Wolchok, 2016). The immune checkpoints refer to a subset of inhibitory signaling pathways presenting in the immune response. Under normal circumstances, the immune checkpoints can maintain immune tolerance by preventing the autoimmune response. However, due to tumor attack, the activation of immune checkpoints can inhibit autoimmunity and facilitate tumor cell growth and escape (Pardoll, 2012). The concept of the immune checkpoint was first proposed in a review article in 2006 by James P. Allison, winner of the 2018 Nobel Prize in Physiology or Medicine (Korman, Peggs, & Allison, 2006). Recently, checkpoint inhibitors (CIs) have completely rewritten the history of tumor immunotherapy and obtained regulatory approval for many other advanced cancer treatments (Cella et al., 2016; Ribas et al., 2013; Rizvi et al., 2015). These exciting results have raised interest in investigating whether these drugs are also effective in the field of brain tumors.

As a family of carbohydrate‐binding proteins, galectins are characterized by their β‐galactoside‐binding affinity and the presence of an evolutionarily conserved sequence: the carbohydrate recognition domain (CRD; Barondes et al., 1994). Galectin‐9 (Gal9) is an important member of the galectin family, it is expressed on lymphocytes and some other cell types (Wada & Kanwar, 1997). Many studies have demonstrated that galectins not only participate in many physiological processes such as brain development (Imaizumi et al., 2011), angiogenesis (Markowska, Liu, & Panjwani, 2010), T‐cell homeostasis (Rabinovich & Toscano, 2009), and feto‐maternal tolerance (Blois et al., 2007) but also participate in tumor progression, immune escape, and tumor angiogenesis (Méndez‐Huergo, Blidner, & Rabinovich, 2017). At the same time, there is an increasing evidence that Gal9 demonstrates as a potential prognostic biomarker and a promising treatment target for certain malignancies (Heusschen, Griffioen, & Thijssen, 2013).

T‐cell immunoglobulin mucin 3 (TIM3) as a Th1‐specific cell surface protein can mediate macrophage activation, inhibit Th1‐mediated immune responses, and promote immune tolerance (Sakuishi et al., 2013). Blocking TIM3 can inhibit tumor growth by enhancing antitumor immunity to diseases such as prostate cancer and hepatocellular carcinoma (HCC; Das, Zhu, & Kuchroo, 2017; Ji et al., 2018; Wu et al., 2017). Gal9 binds to TIM3 to induce Th1 cells death (Zhu et al., 2005). Many studies have found that blocking the Gal9/TIM3 pathway can restore tumor‐infiltrating lymphocyte function (Lemke et al., 2012; Wainwright et al., 2014).

However, as the specific mechanism of Gal9 in GBM remains unclear, an in‐depth study of the biological processes of Gal9 in antitumor immunity will provide a molecular basis for targeted Gal9 therapy. In this study, we evaluated the expression status of Gal9 and its associated biological processes and prognosis by analyzing RNA‐seq data and clinical data from two databases, with the hope of obtaining a comprehensive understanding of Gal9 and new findings in its use for the treatment of GBM.

## 2 METHODS

### 2.1 Sample collection

Gene expression profiling data sets on 325 glioma patients (WHO II‐IV) were gained from the Chinese Glioma Genome Atlas (CGGA, http://www.cgga.org.cn); the data sets from an additional 702 glioma patients (WHO II‐IV) were acquired from the Cancer Genome Atlas (TCGA, http://cancergenome.nih.gov/). Clinical tissue microarray (TMA) and image data (including 50 patients with glioma and 2 epilepsy patients) were obtained from the Department of Neurosurgery, Tianjin Medical University General Hospital (Figure S1). All patients were histologically graded on the basis of the 2016 World Health Organization (WHO) classification of neurological tumors. Written informed consents were provided. The study was conducted in conformity with the principles of the Helsinki Declaration and approved by the Ethics Committee of the General Hospital of Tianjin Medical University.

### 2.2 Immunohistochemistry

Paraffin‐embedded TMAs were dewaxed. After antigen retrieval (treated in 10 mmol/L citrate buffer for 20 min at 92‐98°C), sections were cleared of endogenous peroxidase activity by incubation with 0.3% hydrogen peroxide for 15 min and blocked with goat serum (Solarbio, China) for 30 min at 37°C. They were then incubated with Gal9 antibodies (1:100 dilutions) at 4°C overnight. Then, the TMAs were performed using the DAB Kit (ZSGB‐BIO, China) and counterstained by hematoxylin. The TMAs were examined using an olympus VANOX microscopy.

### 2.3 Statistical analysis

The statistical analysis of most data was performed using R (3.4.2) and public bioinformatics analysis websites, Gene Expression Profiling Interactive Analysis (GEPIA, http://gepia.cancer-pku.cn), The Database for Annotation, Visualization and Integrated Discovery (DAVID, https://david.ncifcrf.gov), and MORPHEUS (https://software.broadinstitute.org/morpheus/). Statistical analysis were also performed using GraphPad Prism 6.0 (GraphPad, La Jolla, CA). *p* < .05 was considered statistically significant.

## 3 RESULTS

### 3.1 Gal9‐related expression profile and clinical outcome in glioma

**Figure 1:**
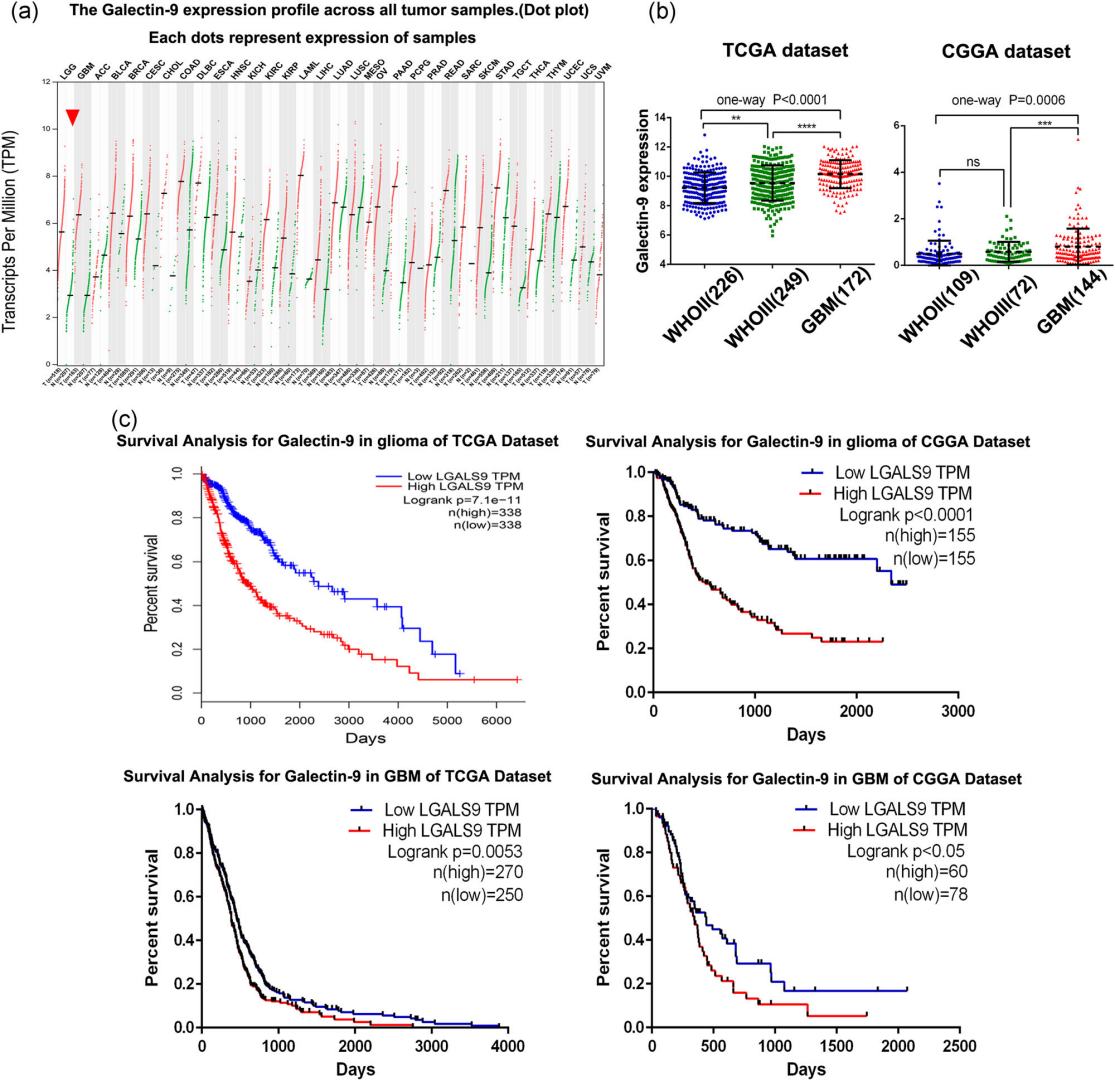
Gal9‐related expression profile and clinical outcome in glioma. (a) Gal9 expression profiles across all tumor samples in the TCGA data set. (b) Compared with LGG, Gal9 was significantly increased in GBM in the CGGA and TCGA data sets. (c) Kaplan–Meier survival analysis showed that patients with Gal9 overexpression had a significantly shorter overall survival in glioma. *, **, and *** indicate *p* < .05, *p* < .01, and *p* < .0001, respectively. CGGA, Chinese Glioma Genome Atlas; Gal9, Galectin‐9; GBM, glioblastoma multiforme; LGG, lower‐grade glioma; TCGA, The Cancer Genome Atlas

To characterize the expression pattern of Gal9 in glioma, we examined the RNA‐sequencing data of glioma from the CGGA and TCGA databases. Similarly, in glioma and many malignancies, Gal9 is more highly expressed in tumor tissues compared with normal tissues (Figure 1a). Compared with lower‐grade glioma (LGG; WHO grade II and WHO grade III), GBM (WHO grade IV) showed the highest Gal9 expression in both databases (Figure 1b). To investigate the effects of Gal9 on glioma survival, we collected 325 glioma patients (included 144 GBM patients) from the CGGA database and 676 glioma patients (included 539 GBM patients) from the TCGA database. As shown in Figure 1c, patients with a high Gal9 expression survived significantly shorter than patients with a low expression when considering all grades of glioma patients. Owing to the heterogeneity of gliomas, we obtained similar results when only considering patients with GBMs (Figure 1c). The results above reflect that Gal9 is strongly associated with the prognosis of GBM patients.

### 3.2 Gal9 was enriched in glioblastoma and could predict worse survival in glioma

**Figure 2:**
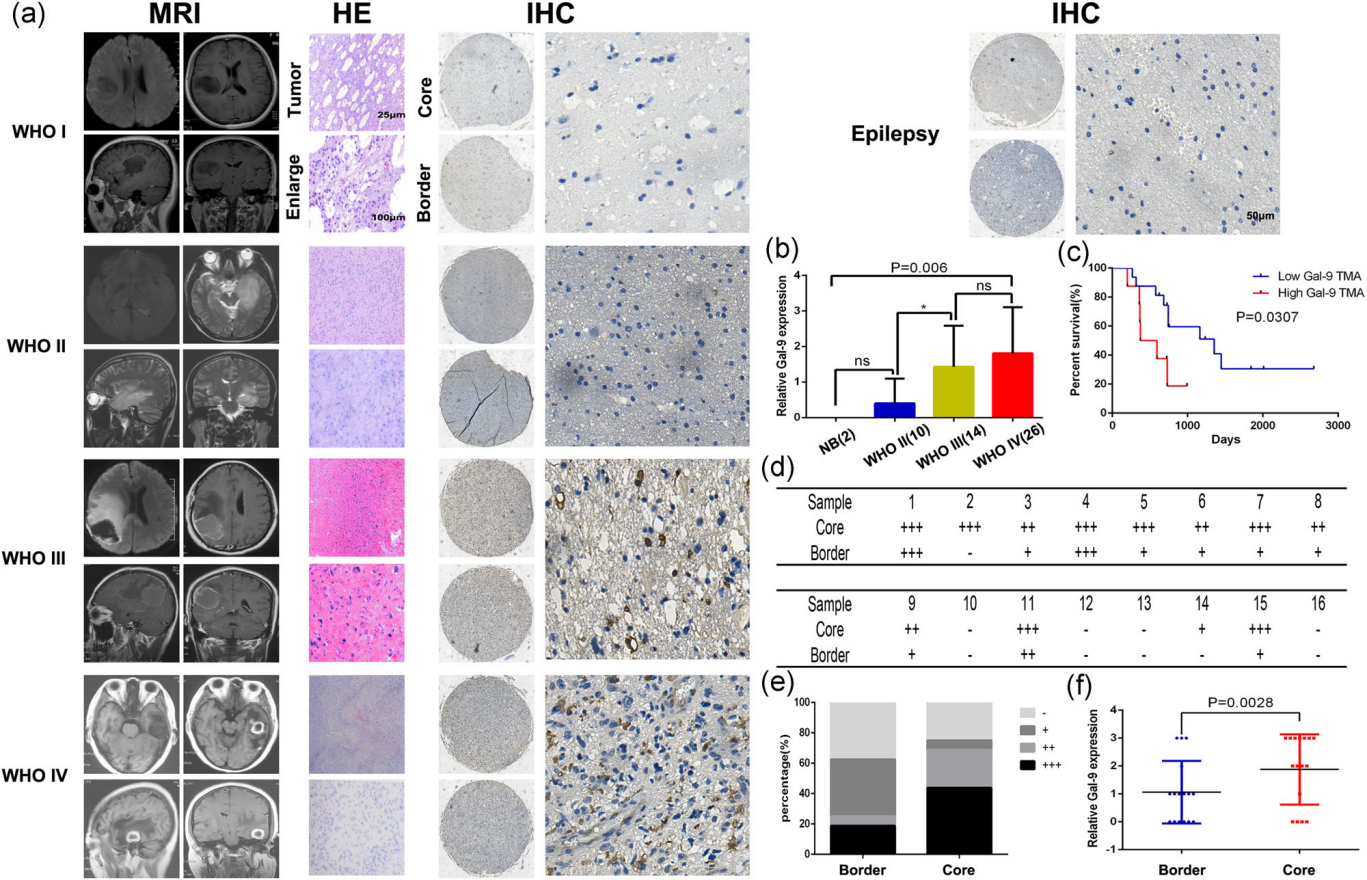
Gal9 was enriched in glioblastoma and could predict worse survival in glioma. (a) MRI images of four glioma patients with representative images of HE and IHC of Gal9; and IHC of Gal9 of one epilepsy patient. (b) Gal9 expression was upregulated in GBM samples compared with LGG and normal brain tissues using IHC by TMA data (*n* = 52). (c) Kaplan–Meier representation of the overall survival of the patients groups with high or low Gal9 expression. (d–f) IHC analysis of Gal9 expression in 16 pairs of core/border GBM tissues. The results of the IHC analysis were revealed in (e) and (f). The intensity of immunostaining was graded as follows: −  and 0, negative; + and 1, weakly positive; ++ and 2, moderately positive; + + + and 3, strongly positive). Three senior pathologists, who were blind to the clinical data, independently scored the TMAs. Gal9, Galectin‐9; GBM, glioblastoma multiforme; HE, hematoxylin‐eosin; IHC, immunohistochemical staining; MRI, magnetic resonance imaging; TMA, tissue microarray; WHO, World Health Organization

At the proteome level, TMA data of brain tumors from Tianjin Medical University General Hospital showed that Gal9 was enriched in GBM (Figure 2a,b) and Gal9 was more highly expressed in the core than in the border of tumors in GBM patients (Figure 2d–f). Likewise, glioma patients with high expressions (+ +/+ + +) of Gal9 had significantly shorter overall survival than those with low expressions (−/+) of Gal9 (Figure 2c). These results indicate that Gal9 plays a key role in the malignant progression of GBM and is closely related to glioma patients’ prognosis.

### 3.3 Gal9 is a novel biomarker of the mesenchymal subtype GBM

**Figure 3:**
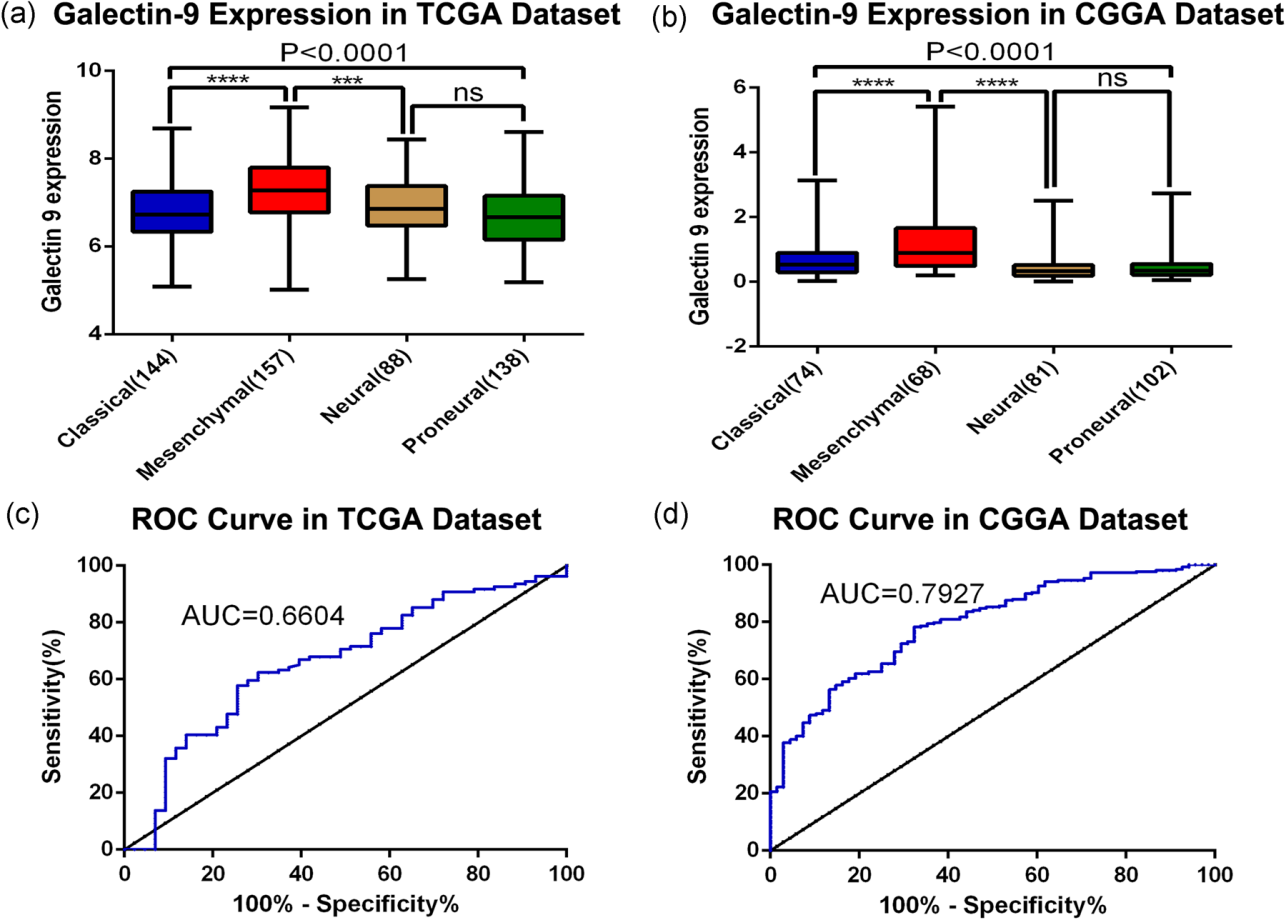
Gal9 is a novel biomarker of the mesenchymal subtype GBM. (a, b) Gal9 expression pattern in different subtypes in the CGGA and TCGA data sets. (c, d) ROC curve analysis showed that Gal9 had high sensitivity and specificity for predicting the mesenchymal subtype in the CGGA and TCGA databases. GBM, glioblastoma multiforme; AUC, area under the curve; CGGA, Chinese Glioma Genome Atlas; Gal9, Galectin‐9; ROC, receiver operating characteristic; TCGA, The Cancer Genome Atlas

To determine the molecular expression pattern of Gal9, we investigated the distribution of Gal9 of different molecular subtypes in the CGGA and TCGA data sets. When compared with the other subtypes, Gal9 was dramatically upregulated in the subtype of mesenchymal GBM (Figure 3a,b). To further validate this, receiver operating characteristic curves for Gal9 expression and for the mesenchymal subtype were performed. The area under curve reached to 79.3% and 66.0% in the CGGA and TCGA data sets, separately (Figure 3c,d). These results revealed that Gal9 was highly and specifically enriched in mesenchymal subtype. So, we inferred that Gal9 may play important biological functions in this subtype.

### 3.4 Gene ontology analysis of Gal9‐associated genes in mesenchymal GBM

**Figure 4:**
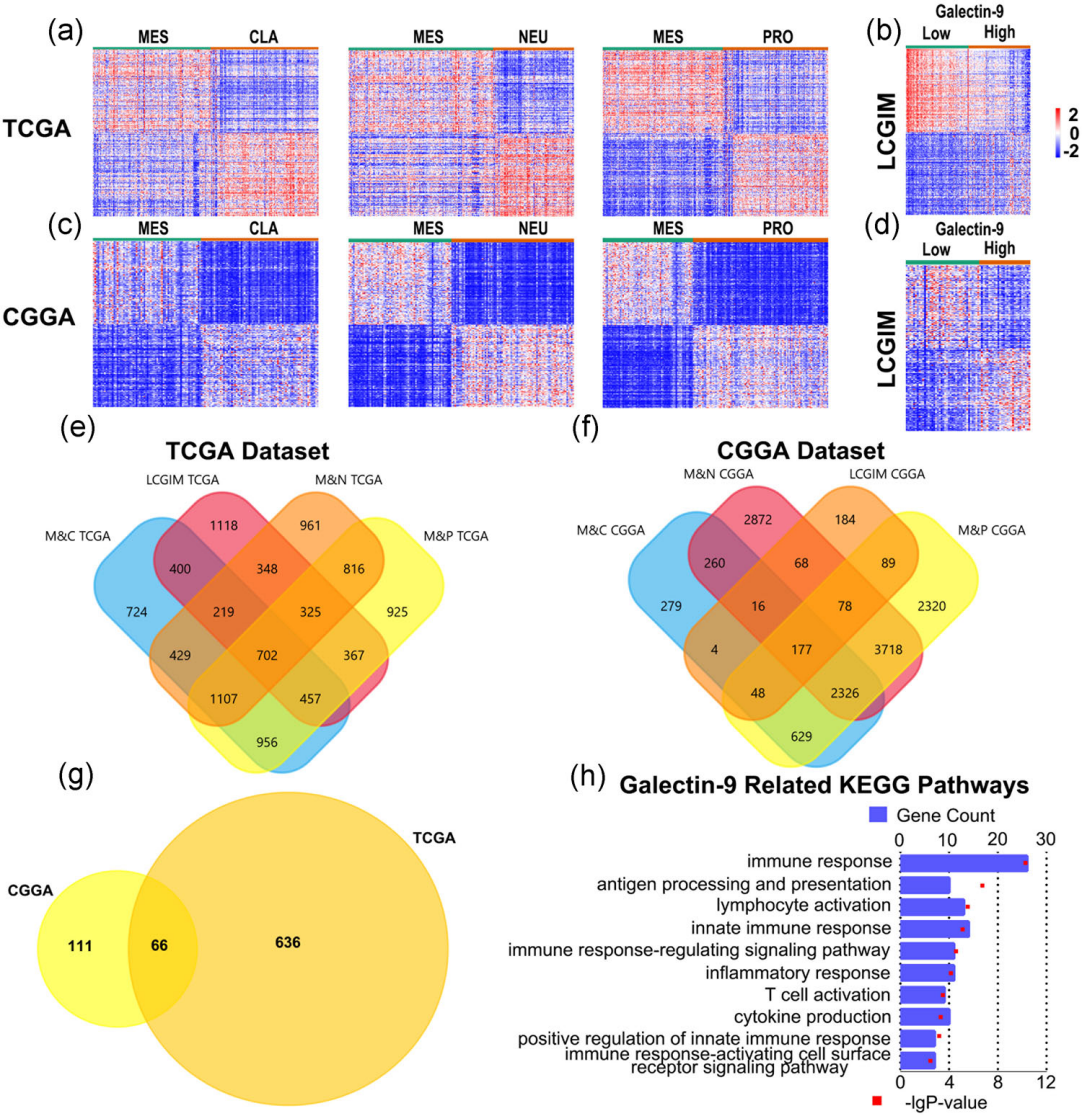
Gene ontology analysis of Gal9‐associated genes in mesenchymal GBM. (a, c) Heat maps display the DEGs between the MES and CLA, NEU, and PRO subtypes of GBM in the CGGA and TCGA data sets. (b, d) Heat map displays the DEGs between the Gal9^high^ and Gal9^low^ groups in MES GBM. (e, f) Venn graphs reveal 177 and 702 overlapping genes specific to the Gal9‐correlated GBM in the CGGA and TCGA data sets, respectively. (g) A comparison of the 177 and 702 genes revealed 66 overlapping genes specific to the Gal9‐correlated GBM. (h) Gene ontology analysis showed that Gal9 was mostly involved in immune response and lymphocyte activation in the CGGA and TCGA databases. CGGA, Chinese Glioma Genome Atlas; CLA, classical; DEG, differentially expressed gene; Gal9, Galectin‐9; GBM, glioblastoma multiforme; KEGG, Kyoto Encyclopedia of Genes and Genomes; MES, mesenchymal; NEU, neural; PRO, proneural; TCGA, The Cancer Genome Atlas

We have studied the potential biological processes, in which Gal9 engaged in mesenchymal GBM. First, the differentially expressed genes (DEGs) between the mesenchymal subtype and the other subtypes of GBM were determined when *p* ≤ .01 (Figure 4a,c). Next, we selected 664 and 3936 DEGs that were highly correlated with Gal9^high^ patients (classified by the mean value of Gal9 mRNA expression) of mesenchymal GBM in the CGGA and TCGA data sets, respectively (Figure 4b,d), and named LGALS9‐correlated genes in mesenchymal (LCGIM) GBM. After mixing the above DEGs, 177 and 702 overlapping genes were obtained in the CGGA and TCGA data sets, respectively (Figure 4e,f). Finally, we compared the CGGA and TCGA data sets to identify the overlapping DEGs, yielding a total of 66 genes (Figure 4g). These genes were further analyzed by Gene ontology (GO) analysis on the DAVID website. We found that Gal9 was mainly involved in immune response and lymphocyte activation (Figure 4h).

### 3.5 The relationship between Gal9 and immune activities in GBM

**Figure 5:**
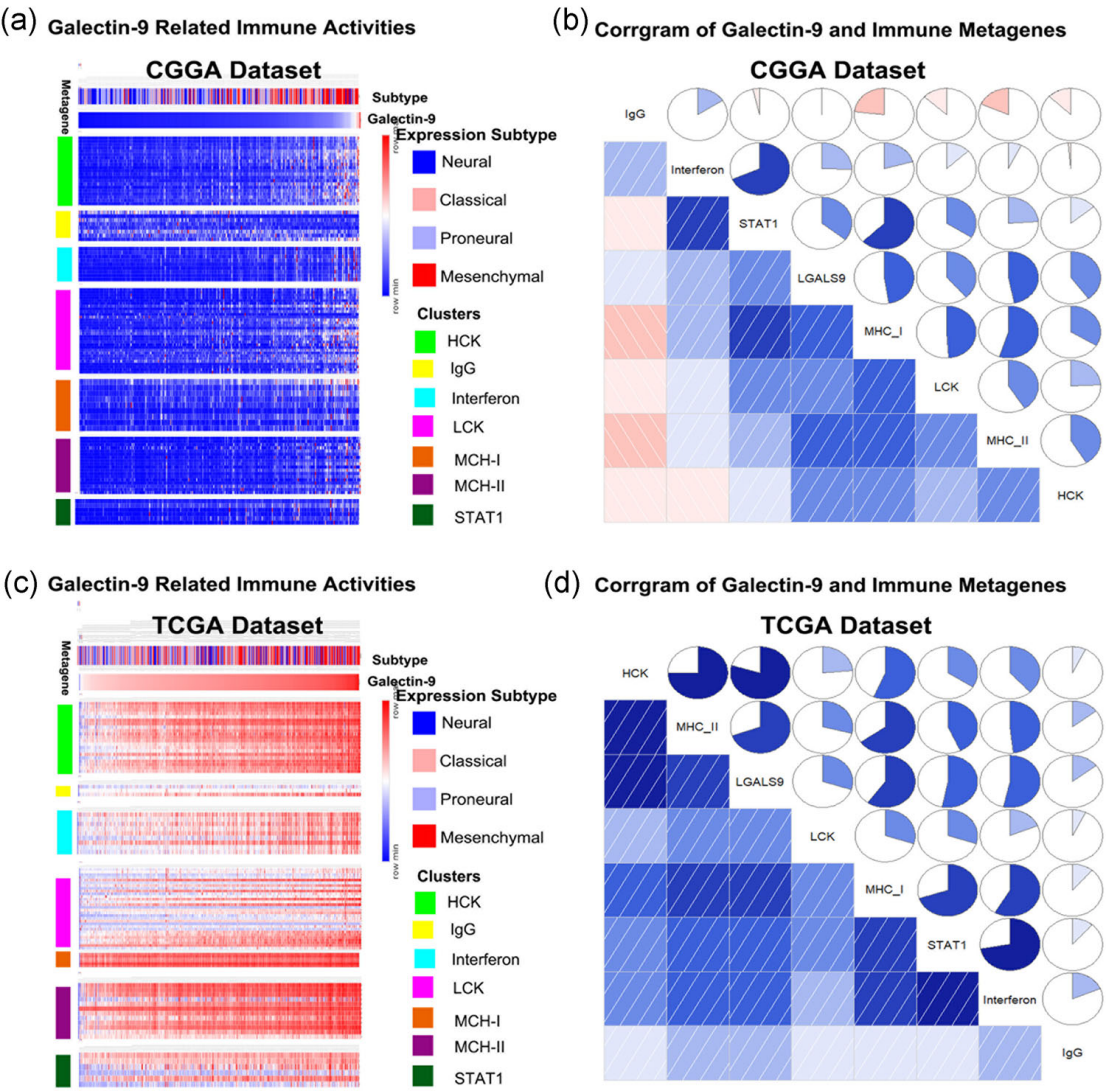
The relationship between Gal9 and immune activities in GBM. (a, c) Heat map represents different types of immune activities in the CGGA and TCGA data sets. (b, d) The relationship between Gal9 and immune activities in GBM. The correlation between Gal9 and other functions was analyzed by Correlation analysis. CGGA, Chinese Glioma Genome Atlas; Gal9, Galectin‐9; GBM, glioblastoma multiforme; IgG, immunoglobulin G; MCH‐I, major histocompatibility complex class I; STAT1, signal transducer and activator of transcription 1; TCGA, The Cancer Genome Atlas

To further explore the specific immune response of Gal9, we selected seven‐gene sets that represent different types of immune response functions and defined them as metagenes (Rody et al., 2009). As shown in Figure 5a,c, most of the metagenes were positively correlated with Gal9 expression except for IgG. Among them, lymphocyte‐specific kinase (LCK), hemopoietic cell kinase (MCK) and Major histocompatibility complex class I (MHC‐I) had the highest positive correlations. To further verify the above results, we obtained corrgrams based on the Pearson's r values between Gal9 and seven metagenes (Figure 5b,d). These results indicated that, in GBM, the macrophage‐ and T‐lymphocyte‐mediated immune responses are closely related to Gal9 but B‐lymphocyte‐mediated immune response has little relationship with Gal9.

### 3.6 Gal9 positively correlate with M2 tumor‐associated macrophages in GBM

**Figure 6:**
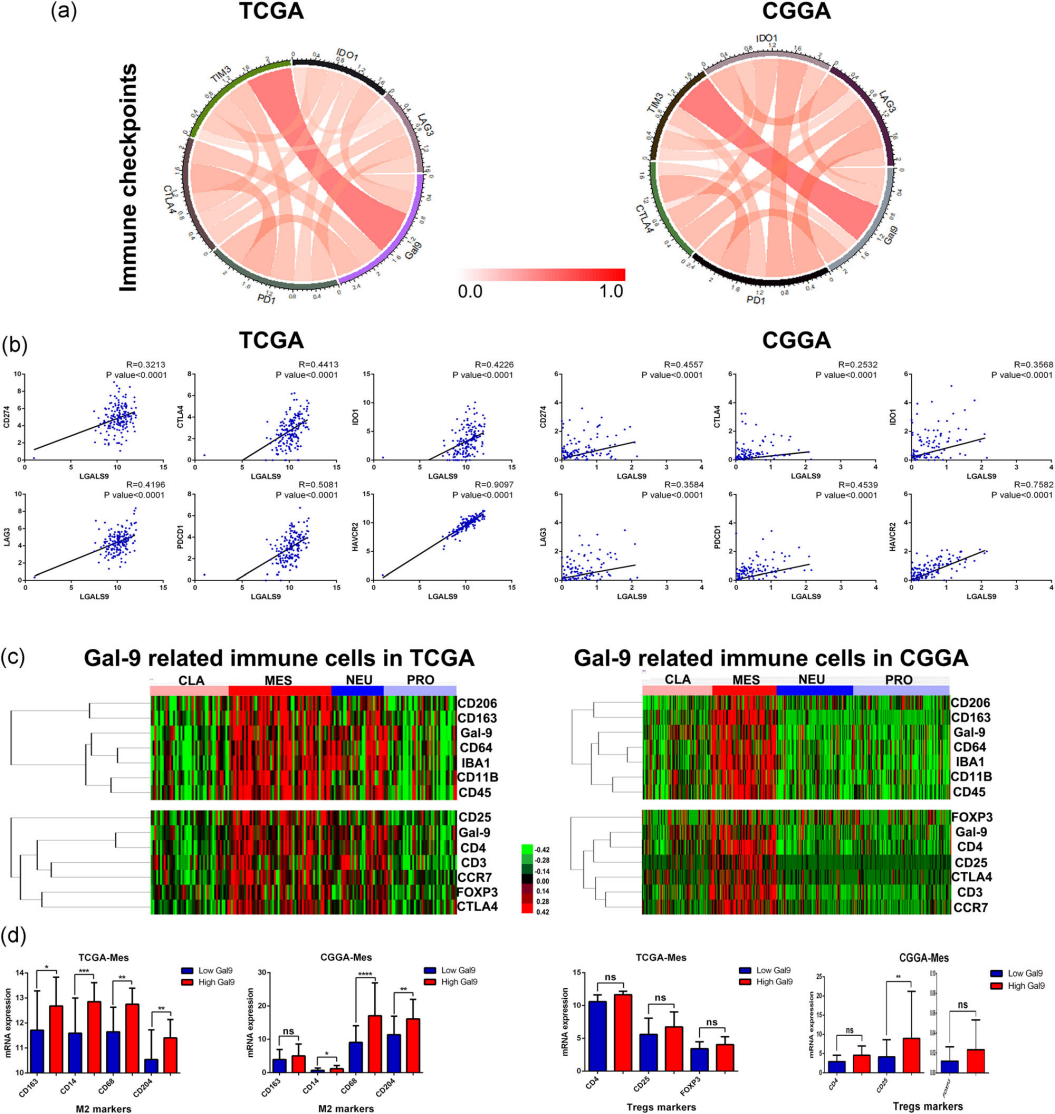
Gal9 positively correlated with M2 tumor‐associated macrophages in GBM. (a,b) Pearson correlation analysis revealed that Gal9 was significantly correlated with immune checkpoint molecules. (c,d) Cluster analysis and Pearson correlation analysis showed that Gal9 was closely related to M2 tumor‐associated macrophages but had little to do with Treg cells in mesenchymal GBM. CGGA, Chinese Glioma Genome Atlas; CLA, classical; Gal9, Galectin‐9; GBM, glioblastoma multiforme; MES, mesenchymal; NEU, neural; PRO, proneural; TCGA, The Cancer Genome Atlas

Pearson correlation analysis showed that Gal9 was notably associated with immune checkpoint molecules including CD274, CTLA4, IDO1, LAG3, PDCD1, and TIM3 (Figure 6a,b). To further investigate the underlying mechanism of Gal9 in antitumor immune responses, we performed cluster analysis and Pearson correlation analysis on key markers of tumor‐associated macrophages and Treg cells. The analysis found that in mesenchymal GBM, Gal9 is closely related to M2 tumor‐associated macrophages but has little to do with Treg cells (Figure 6c,d). Taken together, we hypothesized that Gal9 may exert tumor immunosuppression or immune escape through M2 tumor‐associated macrophages.

## 4 DISCUSSION

Except for tissue invasion, angiogenesis, local tissue hypoxia, and necrosis it can be noted that evasion of innate and adaptive antitumor immune responses also promotes GBM pathogenesis (Zeng et al., 2013). Tumor‐associated local and systemic immunosuppression has gathered researchers’ significant interest. Immunological CIs have shown encouraging results in some advanced malignancies, making cancer researchers think that whether CI exerts the same effect on GBM; thus, a variety of CIs have been developed, such as ipilimumab, nivolumab and pembrolizumab. At the same time, related clinical trials are actively underway, which provides treatment strategies and hopes for GBM patients (Preusser, Lim, Hafler, Reardon, & Sampson, 2015).

Gal9 is an important member from the galectin family and is also a protein with β‐galactoside‐binding affinity. There are research suggesting that Gal9 plays a significant role in tumor biology (Zhou et al., 2018). Early studies have shown that as a TIM3 ligand, Gal9 could bind to TIM3 and downregulate Th1‐type immune response (Zhu et al., 2005). Similarly, Gal9 also promotes immune dysfunction in HCC via the Gal9/TIM3 interaction (Li et al., 2012). The latest study found that Gal9/TIM3 pathway is one of the key mechanisms of immune escape in AML (acute myeloid leukemia; Gonçalves et al., 2017). Recent studies have also found that overexpression of Gal9 can promote macrophages regulation from M1 to M2 and is closely related to TGF‐β, IL‐10, and Stat3 activities (Lv, Bao, & Li, 2017). Recently, increasing attention has been paid to the role of Gal9 in cancer patients. However, the prognostic value and mechanism of Gal9 in GBM remains unclear.

To date, this is the first comprehensive exploration of Gal9 expression, genetic characteristics, and prognostic value in glioma. In this study, we included 1027 glioma patient samples from the CGGA and TCGA databases. Through a series of bioinformatics analyses, Gal9 was closely linked to tumor grade, with the highest expression found in GBM, and the expression of mesenchymal GBM was higher than those of the other subtypes. Patients with gliomas with a high Gal9 expression had significantly lower survival rates than patients with low Gal9 expression. At the same time, this prognostic value was also observed in GBM patients. The above results indicate that Gal9 is involved in the malignant biological process of GBM.

In addition, by analyzing the relationship between Gal9 and immune function‐related gene sets, we found that Gal9 expression was positively correlated with immune checkpoint molecules and M2 tumor‐associated macrophage markers. Gal9 is likely to exert tumor immunosuppression or immune escape through M2 tumor‐associated macrophages.

On the basis of the above results, we conclude that the Gal9/TIM3 pathway maybe one of the key pathways for immune‐related genesis of GBM cells. Blocking the Gal9/TIM3 pathway may infect the malignant progression of glioma, thereby effectively improving the prognosis of patients with GBM.

## CONFLICT OF INTERESTS

The authors declare that there is no conflict of interests.

## Supporting information


**Supplementary Figure 1** Clinical tissue microarray and image data (including two epilepsy patients and 50 patients with glioma) were acquired from the Department of Neurosurgery, Tianjin Medical University General Hospital.Supporting information
