# Supplementary figures and images for: Molecular and clinical characterization of Galectin‐9 in glioma through 1,027 samples
Source: J Cell Physiol. 2019 Oct 14;235(5):4326–34. doi: 10.1002/jcp.29309 (PMC7028024; doi:10.1002/jcp.29309)

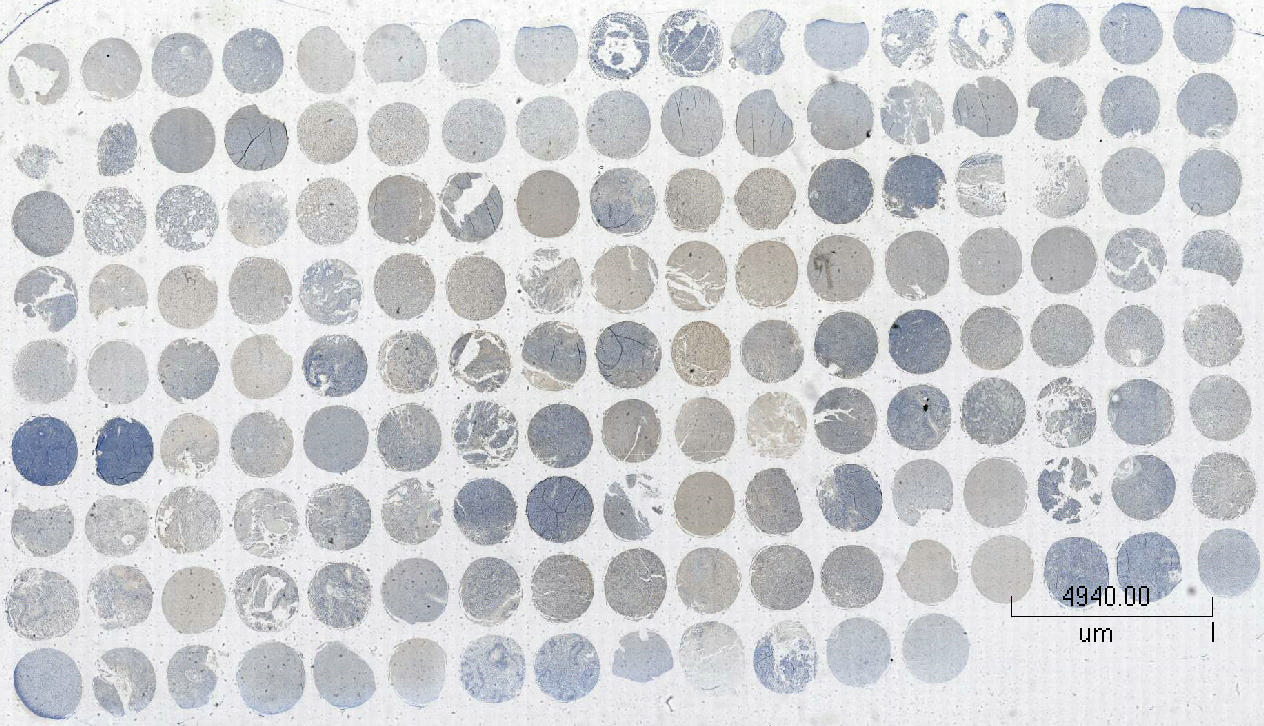

Supplement: Supplementary file 1 — Supplementary Figure 1 Clinical tissue microarray and image data (including two epilepsy patients and 50 patients with glioma) were acquired from the Department of Neurosurgery, Tianjin Medical University General Hospital.Supporting information [file JCP-235-4326-s001.tif]
